# Tumor microenvironment heterogeneity in bladder cancer identifies biologically distinct subtypes predicting prognosis and anti-PD-L1 responses

**DOI:** 10.1038/s41598-023-44028-3

**Published:** 2023-11-10

**Authors:** YaFei Li, Yi Liu, Zhengjun Kang, Jianhua Guo, Nan Liu

**Affiliations:** Department of Urology, the Fifth Affiliated Hospital of Zhengzhou University, Zhengzhou University, Zhengzhou, 450000 Henan Province China

**Keywords:** Cancer microenvironment, Cancer models, Tumour heterogeneity, Tumour immunology, Urological cancer

## Abstract

Bladder cancer (BCa) is heterogeneous in the tumour microenvironment (TME). However, the role of the TME in BCa in modulating the response to immunotherapy has not been fully explored. We therefore analysed fractions of immune cells using CIBERSORTx and clustered BCa into subtypes. We also analyzed weighted correlation networks to generate immunotherapy-related hub genes that we used to construct a prediction model using multivariate Cox and LASSO regression analyses. We found that BCa comprised three subtypes (C1‒C3). The prognosis of the patients was the most favourable and the response rate to anti-programmed death ligand 1 (PD-L1) was the highest in C1 among the three subtypes. Immune cells, including CD8^+^, CD4^+^ memory activated, and follicular helper T cells, activated NK cells, and M1 macrophages infiltrated the C1 subtype. The C2 subtype was enriched in M0 macrophages and activated mast cells, and the C3 subtype was enriched in B and resting immune cells. Mechanistically, the enhanced immunogenicity of subtypes C1 and C2 correlated positively with a higher response rate, whereas the dysregulated ECM-related pathways in the C2 subtype and glycolytic and fatty acid metabolic pathways in the C3 subtype impaired the responses of patients to anti-PD-L1 therapy. We also constructed a TME-related signature based on 18 genes that performed well in terms of overall survival. In conclusion, we determined prognoses and anti-PD-L1 responses by analysing TME heterogeneity in BCa.

## Introduction

Bladder cancer (BCa) is the most common malignancy of the urinary system and the 10^th^ most prevalent cancer worldwide^1^. Non-muscle invasive bladder cancer (NMIBC) has a high incidence of recurrence. At diagnosis, 70–80% of patients have NMIBC, with a 5-year overall survival (OS) > 85%. However, the recurrence rate of NMIBC is 60‒70%^2^ and 20‒25% of NMIBC progress to MIBC, which has a the 5-year BCa survival rate of < 50%^3,4^. The heterogeneity of BCa results in complex treatment strategies, but current classifications based on TNM or grade/stage meet the need for more targeted and personalised approaches. Therefore, a better understanding of the mechanisms underlying the development and progression of BCa is necessary to identify novel management strategies.

The tumour microenvironment (TME) consists of malignant and non-malignant cells, and the interplay of components in the TME can regulate the progression of cancer and its response to drugs^5^. The introduction of agents that block this interaction has significantly improved the outcomes of patients with BCa. For example, programmed death ligand 1 (PD-L1) is ubiquitously expressed in haematopoietic, endothelial, epithelial, and immune cells. Programmed death ligand 1 PD-L1 is mainly responsible for maintaining immune homeostasis in non-tumoural tissues^6,7^. However, the PD1/PD-L1 pathway can be hijacked by tumours to induce immune suppression^8^. Immunotherapeutic options targeting these immune checkpoints have significant clinical effects on BCa^9^. Atezolizumab is an antibody directed against PD-L1 that has been approved as a second-line therapy for advanced BCa as it significantly prolongs the survival of patients compared with other second-line regimens^10^. The currently unsatisfactory response rates impede the further application of these agents in BCa. Therefore, appropriate patients with BCa who are sensitive to such agents should be identified or modulated to become more immunogenic and increase the effectiveness of these approaches. This would encourage better understanding of the complex interaction of elements in the context of the TME.

The present study aimed to identify the infiltrative profiles of patients with BCa and reveal the intrinsic molecular features of underlying subtypes. We also constructed a TME-related signature to predict the prognosis of BCa.

## Materials and methods

### Data source and processing

Expression profiles of the IMvigor210 cohort that received atezolizumab therapy (n = 348) were extracted from the IMvigor210CoreBiologies packages^11^, then read counts were normalised by transcripts per million (TPM) via IBOR packages^12^. We classified patients with complete (CR) and partial (PR) responses as responders, and those with stable (SD) and progressive (PD) disease as non-responders. We also extracted PD-L1 immunohistochemical expression in immune (ICs) and tumour (TCs) cells from the IMvigor210CoreBiologies packages. Gene expression profiles (n = 408) were downloaded from The Cancer Genome Atlas (TCGA)-bladder urothelial cancer (BLCA) data portal (https://portal.gdc.cancer.gov/) in fragments per kilobase per million (FPKM) that were also converted to TPM values. The somatic variants of the TCGA-BLCA cohort processed by VarScan2 were also downloaded and visualised using the maftools package in R^13^. Expression profiles (n = 165) of the GSE13507 cohort and corresponding clinical data were extracted from the Gene Expression Omnibus (GEO) (https://www.ncbi.nlm.nih.gov/geo/).

### CIBERSORTx analysis

CIBERSORTx is an online machine learning method that can infer cell types from bulk tissue transcriptomes and scRNA-seq data^14^. We used CIBERSORTx to analyse expression profiles of the IMvigor210 and TCGA-BLCA cohorts, which were initially normalised using log2(TPM + 1). The relative abundances of 22 types of immune cells were calculated via 1000 permutations and quantile normalisation. We produced *P* values using permutation tests to assess the reliability of deconvolution, and samples with *P* < 0.05 were further analysed.

### Consensus cluster analysis and principal component analysis

We used ConsensusClusterPlus packages^15^ based on the fraction value of 22 types of immune cells to analyse consensus clusters. The optimal K value was identified using empirical cumulative distribution function (CDF) and delta area plots. We ascertained the robustness of the consensus cluster results using principal component analysis (PCA).

### Single sample gene set enrichment analysis

We extracted 50 pathways associated with amino acid, carbohydrate, energy, lipid, and nucleotide metabolism from the Gene Set Enrichment Analysis (GSEA) database (http://www.gsea-msigdb.org) to identify the metabolic status of the clusters. We then calculated the scores for each pathway in all samples using single-sample (ss) GSEA. Differentially expressed pathways with false discovery rates (FDR) < 0.05 were identified using Linear Models for Microarray Data (Limma) packages.

### Weighted correlation network analysis

We identified hub genes associated with clusters and anti-PD-L1 responses using weighted correlation network analysis (WGCNA). We obtained differentially expressed genes (DEGs) with log fold change (|logFC|) > 1 and FDR < 0.05 between every two clusters using Limma packages. The intramodular connectivity of each gene was then calculated to form a weighted adjacency matrix. The soft power threshold was assessed to render the constructed network scale-free, then transformed into a topological overlap matrix. We categorised DEGs with high correlations into modules and merged those that were similar using a dynamic cut tree. Thereafter, we identified hub genes using correlation analysis between the principal components of each module and clinical parameters. Pathway enrichment of genes extracted from each module were analysed using ClusterProfiler version 4.0^16^. Terms with *P* < 0.05 were deemed significant.

### Prognostic model construction

We used the IMvigor210 cohort as the training set to build the prognostic model, and the TCGA-BLCA and GSE13507 cohorts as the calibration set. Prognostic genes among the DEGs were identified using univariate Cox regression analysis. We also analysed protein–protein interactions (PPIs) to identify hub genes using String (https://cn.string-db.org/). The intersection of PPI and univariate Cox regression analyses was further analysed. We applied LASSO regression analysis to avoid overfitting, followed by tenfold cross-validation. Optimal genes for constructing a prognostic model were identified using multivariate Cox regression analysis. Risk scores for the patients were calculated as:

$$ Risk\;score = \sum xi*\beta i $$

x: the expression of gene; β: the coefficient of gene.

Patients were then divided into high- and low-risk groups according to the median risk score, then we determined the impact of risk scores on the OS of patients with BCa using Kaplan–Meier (K-M) curves.

### Statistical analysis

**Figure 1:**
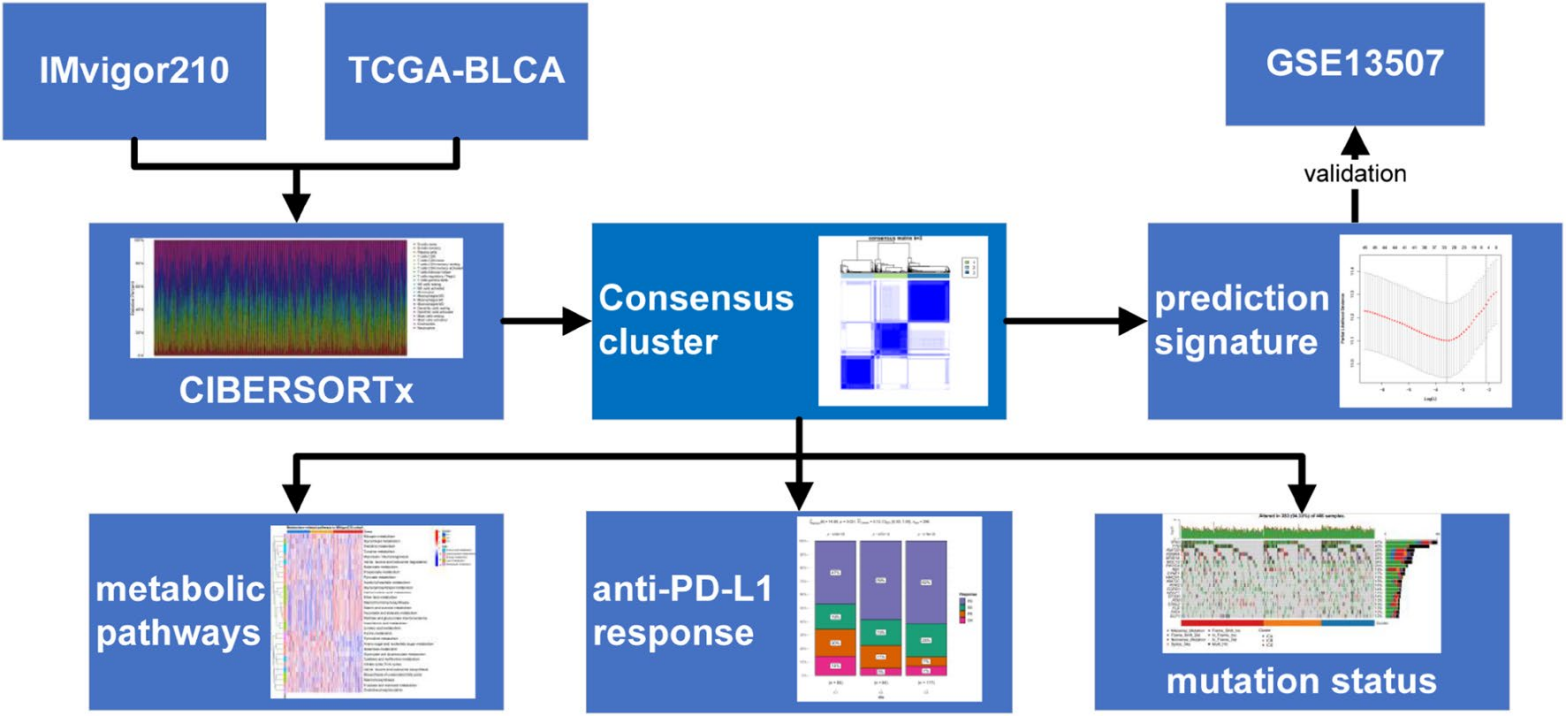
Flow chart of present study.

Clinical data were compared between groups using chi-square or Fisher exact tests. Differences in the expression of PD-L1, TMB, and immune cells were analysed using Wilcoxon rank-sum or Kruskal–Wallis rank-sum tests. Relationships between clusters and groups with OS were determined from K-M survival curves and the significance of differences was determined using log-rank tests. The ability of our model to accurately predicting OS in patients with BCa was determined using receiver operating characteristic (ROC) curves and areas under the curve (AUC). All statistical tests were two-sided, and *P* < 0.05 was considered statistically significant. All data were analysed using R version 4.0.5 (http://www.r-project.org/) with the following Limma, ConsensusClusterPlus, GSVA, survival, and WGCNA packages. Figure 1 shows a flowchart of our study.

## Results

### Immune cell cluster correlated with survival of BCa

**Figure 2:**
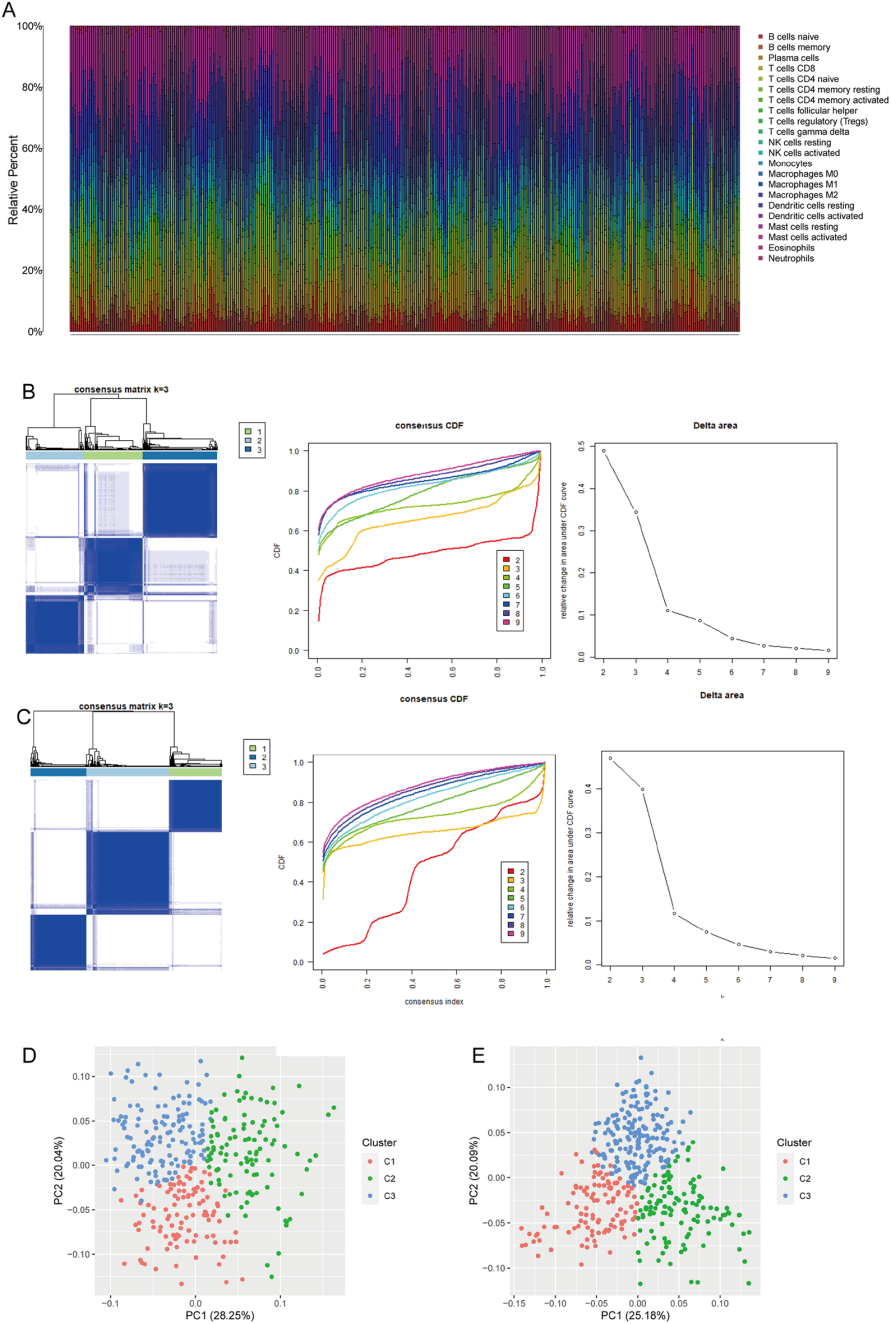
Subtypes of bladder cancer (BCa). **A** Barplot of 22 types of immune cells in BCa shown in different colours. **B** Empirical CDF plot and delta area plot of k = 2 to 9. Consensus score matrix is shown when k = 3 in the IMvigor210 cohort. **C** Principal component analysis divided IMvigor210 cohort into three clusters. **D** Empirical CDF and delta area plots for k = 2 to 9. Consensus score matrix is shown when k = 3 in TCGA-BLCA cohort. **E** Principal component analysis divided TCGA-BLCA cohort into 3 clusters.

We uploaded expression profiles to the online tool CIBERSORTx to infer the fraction of BCa immune cells in the bulk tissue transcriptome (Fig. 2A and Supplementary Fig. 1). Activated mast cells (19.87%) comprised the most prevalent type of immune cells in the IMvigor210 cohort, followed by M2 macrophages (13.70%), and CD8^+^ T cells (9.88%). We then explored the immune infiltration profile of BCa using consensus cluster analysis. Figure 2B shows that patients were clustered into subtypes C1‒C3 based on fractions of 22 types of immune cells. The PCA results revealed the heterogeneity of the subtypes identified by the consensus cluster analysis more clearly, and we found that the three clusters differed (Fig. 2C). Patients in the TCGA-BLCA cohort were divided into three clusters in parallel with the IMvigor210 cohort (Fig. 2D and E).

**Figure 3:**
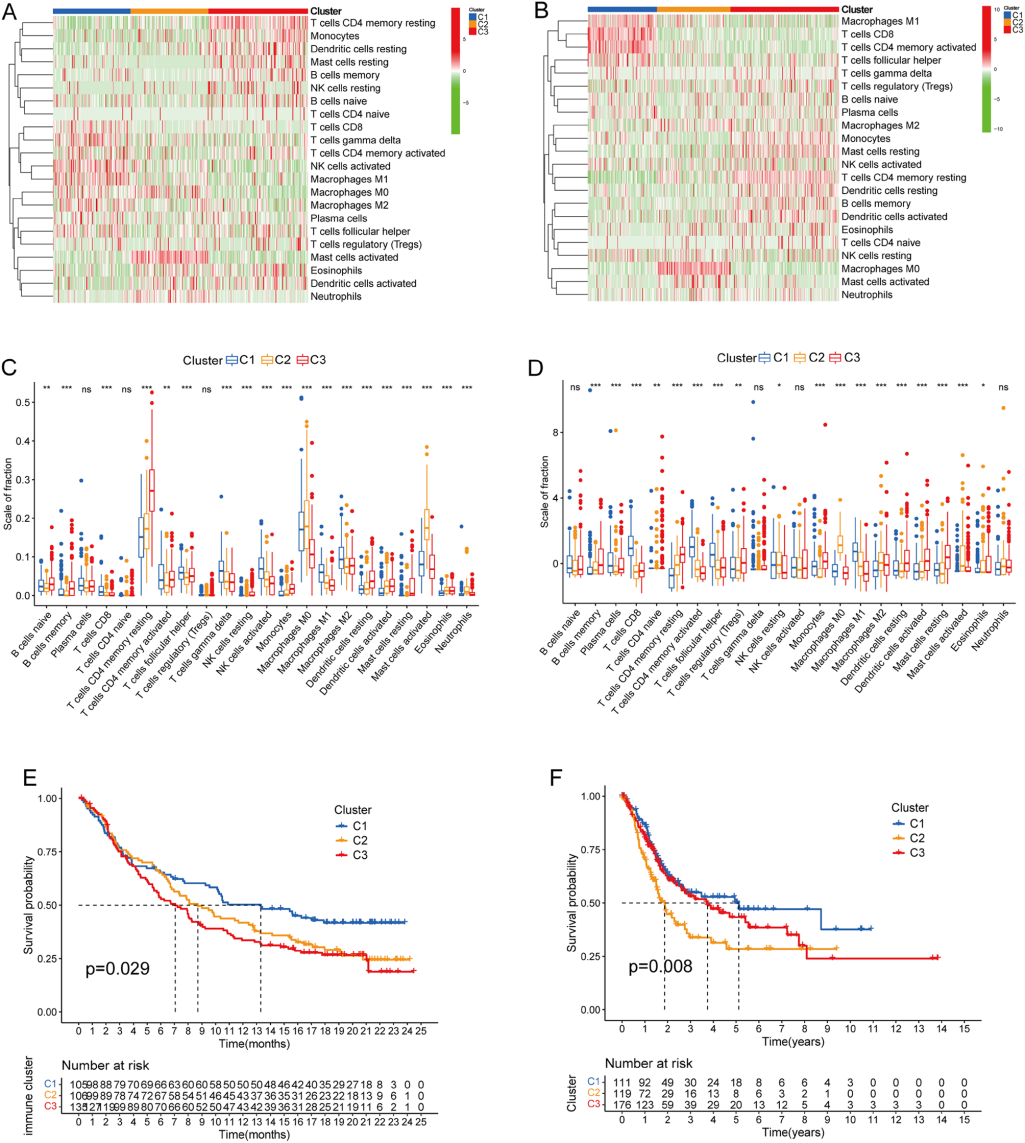
Immune infiltration profiles across clusters. Heatmap of 22 types of immune cells in IMvigor210 (**A**) and TCGA-BLCA (**B**) cohorts. Red and green represent high and low expression, respectively. Distribution of 22 types of immune cells in three subtypes in IMvigor210 (**C**) and TCGA-BLCA (**D**) cohort. Kaplan–Meier curves revealed associations between BCa subtypes and overall survival in the IMvigor210 (**E**) (p = 0.029) and TCGA-BLCA (**F**) cohorts (p = 0.008). *p < 0.05, ^†^p < 0.01, ^‡^p < 0.001; ns, not significant.

The abundance of 22 types of immune cells was similarly distributed across the three clusters in the IMvigor210 and TCGA-BLCA cohorts. The C1 subtype was enriched in CD8^+^, activated memory CD4^+^, and follicular helper T cells, activated NK cells, and M1 macrophages. The C2 subtype was enriched in M0 macrophages and activated mast cells. The C3 subtype was enriched in resting immune and B cells (Fig. 3A‒D). The K-M survival curves in the IMvigor210 cohort revealed that the prognosis was favourable among patients with the C1 subtype, but did not differ between those with the C2 and C3 subtypes (Fig. 3E). The OS was also better for patients with the C1 subtype, than the other two subtypes, whereas the prognosis was the worst for C3 in the TCGA-BLCA cohort (Fig. 3F).

### Association of anti-PD L1 response with subtypes in the IMvigor210 cohort

**Figure 4:**
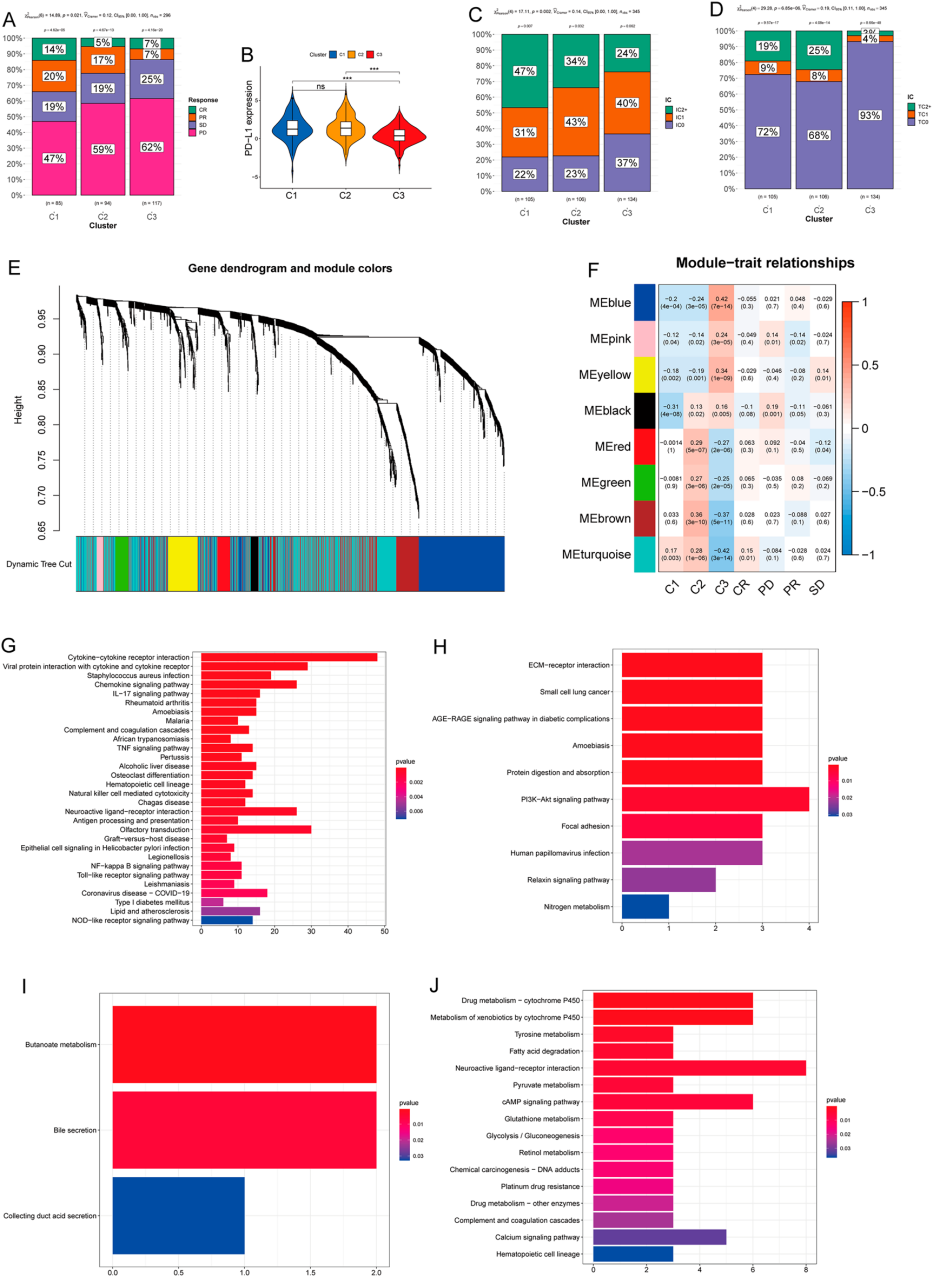
Associations between subtypes and anti-PD-L1 responses. (**A**) Response rates of C1 (34%), C2 (22%), and C3 (14%) subtypes. (**B**) Violin diagrams indicate abundance of PD-L1 mRNA in three subtypes. C3 subtype had the lowest PD-L1 mRNA abundance. (**C**) Immunohistochemical findings of PD-L1 expression in immune cells (ICs) among subtypes IC0 (< 1%), IC1 (≥ 1% but < 5%), and IC2 + (≥ 5%). (**D**) PD-L1 Immunohistochemical findings of PD-L expression in tumor cells (TCs) among subtypes, TC0 (< 1%), TC1 (≥ 1% but < 5%), and TC2 + (≥ 5%). (**E**) Differentially expressed genes divided into eight modules by WCGNA. (**F**) Heatmap of corresponding correlation coefficients and p-values between modules and groups. Kyoto Encyclopedia of Genes and Genomes (KEGG) enrichment analysis based on genes in turquoise (**G**), black (**H**), pink (**I**), and yellow (**J**) modules Terms with p < 0.05 significantly differed.

The response rates of the C1 and C3 subtypes in the IMvigor210 cohort were the highest and lowest, respectively (*P* = 0.021; Fig. 4A). We then found more abundant PD-L1 mRNA in the C1 and C2 subtypes among the three clusters (*P* < 0.001; Fig. 4B). The immunohistochemical expression of PD-L1 in ICs was significantly higher in the C1, than the other two subtypes (*P* = 0.009; Fig. 4C). We also found the least PD-L1 expression in TCs in the C3 subtype (*P* < 0.001; Fig. 4D).

We determined why the response rates differed across clusters by firstly analyzing DEGs in the three clusters and identified 1,787 with |logFC|> 1 and FDR < 0.05. We then categorised these genes into eight modules using WGCNA (Fig. 4E‒F). Genes in the turquoise module correlated closely with immune-related cytokine-cytokine receptor interaction, IL-17 signalling, and TNF signalling pathways (Fig. 4G). The black, red, green, and brown modules were associated with C2 and these genes correlated with ECM-related pathways (Fig. 4H and Supplementary Fig. 2). The black, blue, pink, and yellow modules were associated with upregulated metabolic pathways and correlated positively with the C3 cluster (Fig. 4I and J and Supplementary Fig. 2). Taken together, C1 was strongly immunoreactive, C2 was strongly immunoreactive but had increased invasion and metastasis, and metabolic pathways were the most upregulated in C3. The turquoise module was positively associated with CR, whereas the pink, black, and yellow modules were negatively related to the response. The yellow module was enriched with pathways associated with fatty acid metabolism and glycolysis. Taken together, we found that the ECM- and metabolism-related pathways resulted in impaired responses in clusters 2 and 3.

### Metabolism pathways among subtypes

**Figure 5:**
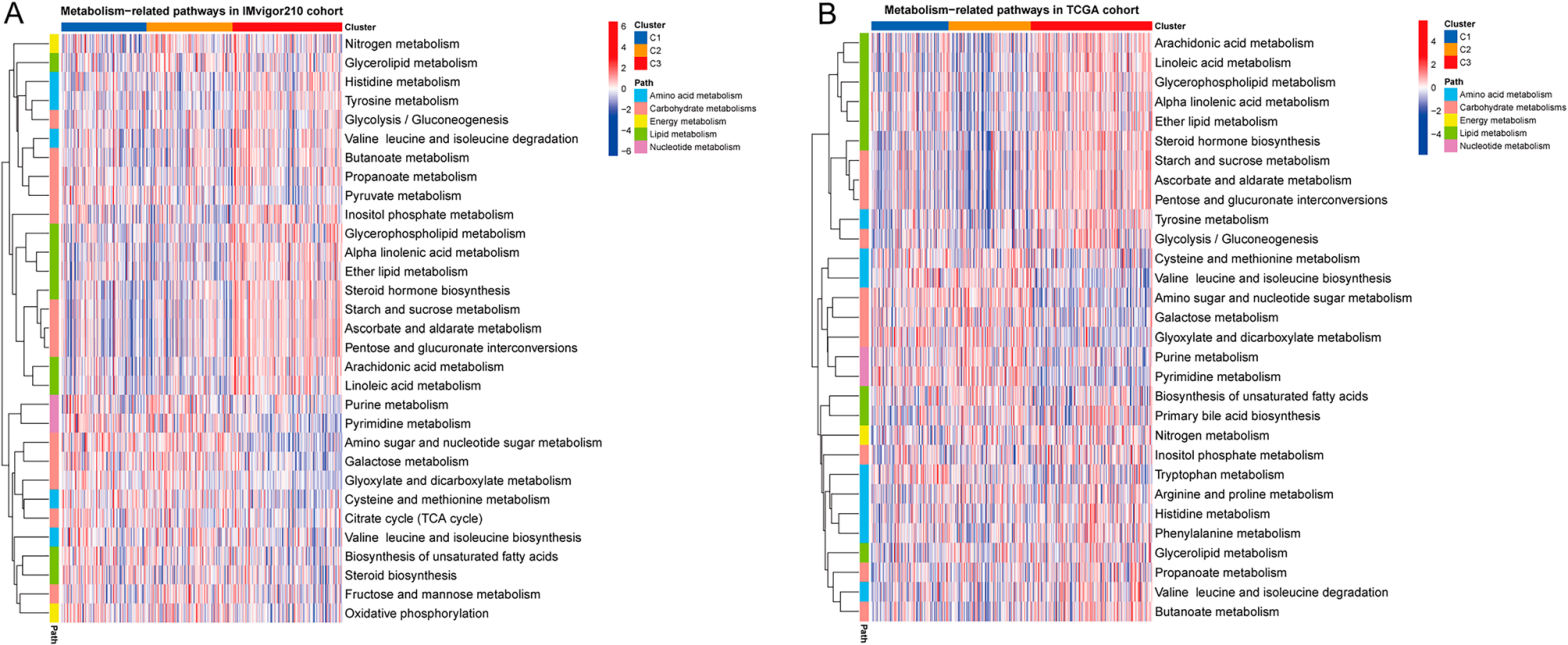
Metabolic pathways among subtypes. Heatmap of differentially expressed terms within five main metabolic pathways in IMvigor210 (**A**) and TCGA-BLCA cohorts (**B**). Red and blue represent high and low expression, respectively.

The WCGNA revealed heterogeneity across subtypes. We therefore quantified the main pathways of amino acid, carbohydrate, energy, lipid, and nucleotide metabolism among the three subtypes using ssGSEA to better understand the effects of metabolism in BCa. The metabolic profiles were similar between the two cohorts. The metabolic features of the C1 and C2 subtypes were similar and distinctly differnt from C3 subtype in both cohorts (Fig. 5A and B). Corresponding to the WCGNA results, mostly lipid and some carbohydrate (glycolysis/gluconeogenesis) metabolism were enriched in C3 subtype. Nucleotide metabolic pathway expression was elevated in subtypes C1 and C2.

### Mutation status in subtypes

**Figure 6:**
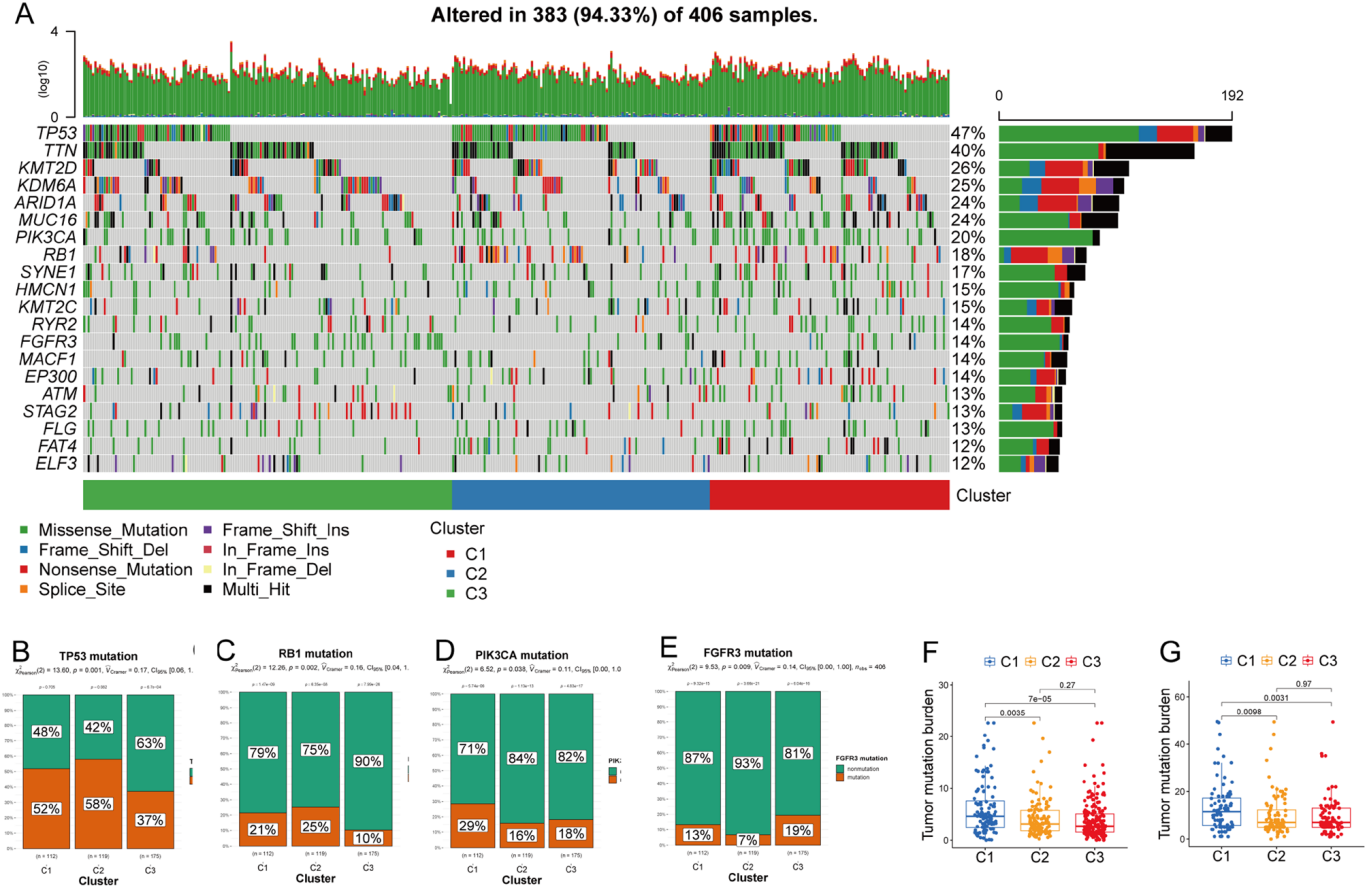
Mutation among three subtypes. (**A**) Waterfall diagram of three subtypes in TCGA-BLCA cohort shows top 20 mutated genes among three subtypes. Mutations in TP53 (**B**) and RB1 (**C**) were more common in C2, whereas those of PIK3CA (**D**) and FGFR3 (**E**) were more prevalent in the C1 and C3 subtypes, respectively. Differences among tumor mutation burdens in three subtypes in TCGA-BLCA (**F**) and IMvigor210 (**G**) cohorts. C1 subtype has highest burden of tumor mutations among three subtypes in both cohorts.

We first visualized the integration status of somatic mutations in BCa from a waterfall diagram,. We found the frequencies of missense mutations, single nucleotide polymorphisms (SNPs), and C > T mutations were the highest in all three subtypes (Fig. 6A and Supplementary Fig. 3). We also analysed the incidence of mutations in frequently mutated genes, including tumour protein 53 (TP53), retinoblastoma (RB1), phosphatidylinositol-4,5-bisphosphate 3-kinase catalytic subunit alpha [(human)] PIK3CA, and fibroblast growth factor receptor 3FGFR3, among the three subtypes. We found that mutations in TP53 (*P* = 0.001) and RB1 (*P* = 0.002) were more common in subtypes C1 and C2 (Fig. 6B and C), whereas PIK3CA was more common in C1 (*P* = 0.038; Fig. 6D), and C3 had more FGFR3 mutations (*P* = 0.009; Fig. 6E). The tumour mutation burden (TMB) of each sample was calculated, and the C1 subtype had the most abundant TMB in the TCGA-BLCA and IMvigor210 cohorts (Fig. 6F and G).

### Construction prognosis signature to predict the survival of BCa

**Figure 7:**
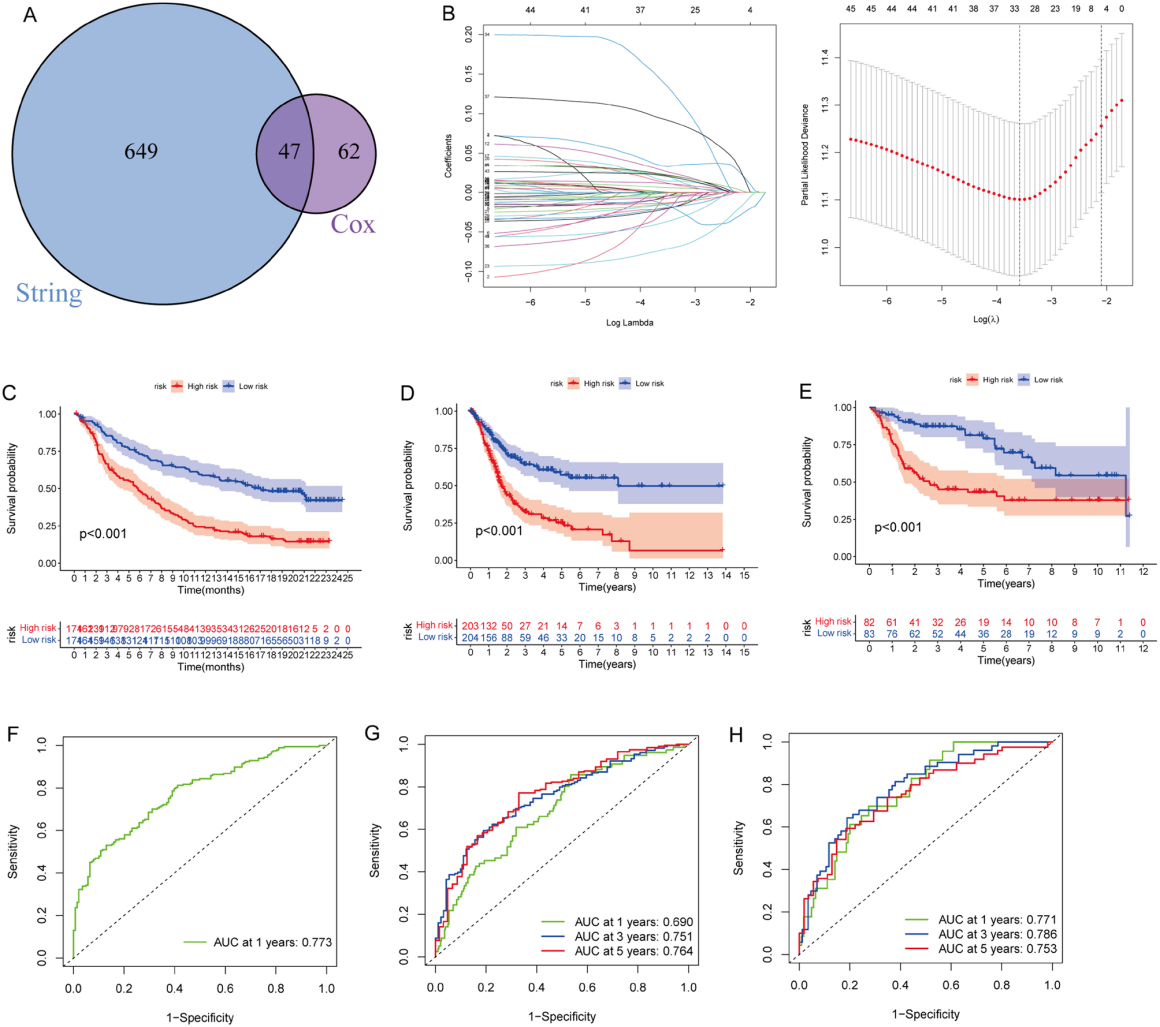
Construction of prediction model in BCa. (**A**) Intersection of string and univariate cox regression analysis. (**B**) LASSO coefficient profiles of each parament and 10-times cross-validation plot of model. The optimum model was obtained when lambda = 0.028. Kaplan–Meier curves revealed associations between BCa subtypes with overall survival in IMvigor210 (**C**), TCGA-BLCA (**D**), and GSE13507 (**E**) cohorts (p < 0.001) for all. (**F**–**H**) ROC curves in IMvigor210, TCGA-BLCA, and GSE13507 cohorts.

We constructed a TME signature to predict BCa cell survival. The IMvigor210 cohort was the training set. Univariate Cox regression analysis associated 109 of 1,787 DEGs with the OS of BCa. We then analysed PPIs that revealed 696 hub genes with confidence > 0.8,among which, 47 were shared between the two sets of results (Fig. 7A). LASSO regression analysis identified 33 genes that we analysed using multivariate Cox regression analysis (Fig. 7B). Eighteen genes were selected to construct the TME signature (Table 1). Risk scores for all the patients were calculated, then the patients were assigned to high- and low-risk groups based on the median risk score. Survival was worse for high-risk patients in the IMvigor210 cohort (*P* < 0.001; Fig. 7C). Survival rates also differed between high-and low-risk patients in the TCGA-BLCA (*P* < 0.001) and GSE13507 cohorts (*P* < 0.001) in the validation sets (Fig. 7C‒E). The TME signature performed well in assessing 1-year OS (AUC = 0.773) in the IMvigor210 cohort (Fig. 7F). The TME signature also performed well in predicting 3- and 5-year OS in the TCGA-BLCA (AUC = 0.690, 0.751, and 0.764 for 1-, 3-, and 5-year OS, respectively) and GSE13507 (AUC = 0.771, 0.786, and 0.753 for 1-, 3-, and 5-year OS, respectively) cohorts in the validation sets (Fig. 7G and H).

**Table 1:** The multivariate Cox regression of 18 genes.

Gene	coef	HR	95CI	*P*
CA12	− 0.083	0.92	0.845–1.002	0.056
GJB2	0.082	1.086	1.005–1.173	0.037
UGT1A10	0.056	1.058	1.017–1.101	0.005
ITIH2	0.013	1.013	0.997–1.029	0.115
KIR3DL3	− 0.027	0.973	0.952–0.995	0.016
CXCL13	− 0.112	0.894	0.849–0.941	< 0.001
SOCS1	− 0.04	0.961	0.920–1.003	0.07
LCT	− 0.032	0.968	0.941–0.996	0.024
CA4	− 0.021	0.979	0.953–1.006	0.133
USP17L1P	0.034	1.035	1.014–1.056	0.001
CHRM2	− 0.019	0.981	0.963–0.999	0.041
ITGA5	0.266	1.305	1.106–1.538	0.002
CD274	− 0.094	0.91	0.803–1.031	0.139
MT1E	0.113	1.119	1.017–1.231	0.021
WNT2	− 0.061	0.941	0.902–0.981	0.004
B3GNT4	0.017	1.017	0.997–1.037	0.105
SPINK14	0.032	1.032	1.012–1.052	0.001
MUC5B	0.029	1.03	1.006–1.055	0.016

### Correlation between TME signature and anti-PD-L1 response

**Figure 8:**
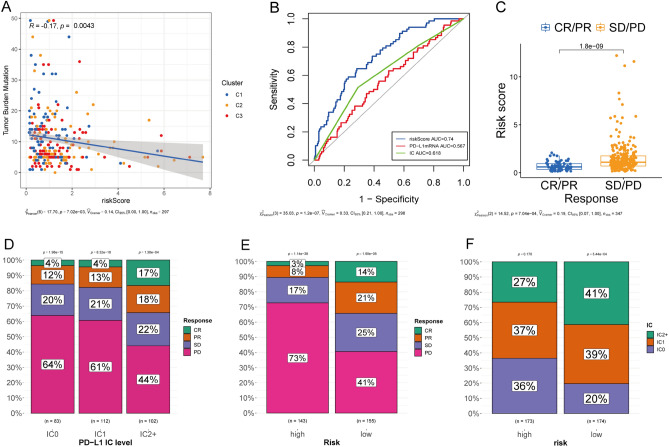
Validation of signature to predict anti-PD-L1 response. (**A**) Risk scores was negatively correlated with tumor mutation burden. (**B**) ROC curves show the abilities of risk scores (AUC = 0.74), PD-L1 mRNA (AUC = 0.567), and PD-L1 IC scores (AUC = 0.618) to predict anti-PD-L1 response. (**C**) Risk scores were higher among non-responders than responders. (**D**) Patients with more abundant PD-L1 ICs had better response rates. (**E**) Response rates in the high *vs*. low risk groups (11% *vs*. 35%). (**F**) Immunohistochemically determined PD-L1 expression in immune cells (ICs) in high- and low-risk groups.

We explored correlations between the TME signature and TMB and found that risk scores negatively correlated with TMB (R = − 0.17, *P* = 0.004; Fig. 8A). The TME signature also predicted responses to anti-PD-L1 therapy, and Fig. 8B shows that its predictive capability (AUC = 0.740) exceeded that of PD-L1 mRNA (AUC = 0.567) and of immunohistochemical findings in ICs (AUC = 0.618). Response rates were significantly higher among patients with IC2^+^ status (Fig. 8C) and in the low-, than in the high-risk group (*P* < 0.001; Fig. 8D and E). Immunohistochemical scores were significantly higher in the low-risk group (*P* < 0.001; Fig. 8F).

## Discussion

Bladder cancer is the most prevalent cancer of the urological system and it is histologically and genomically heterogeneous, which results in highly variable outcomes. Although improved strategies, such as the introduction of Bacillus Calmette-Guerin (BCG) and cisplatin-based chemotherapy, have significantly improved prognosis, that of high-risk, advanced, and metastatic BCa remains unfavourable. Immune checkpoints (ICs) are central mediators of the TME, and IC inhibitors (ICIs) have emerged as new treatment options for patients with BCa. However, a considerable proportion of patients do not respond to ICIs^17^. Selecting an adequate population that responds to ICIs and defining specific biomarkers that can predict the efficiency of ICIs are key to their further application. The TME of BCa varies greatly, and exploring mechanisms leading to distinct responses to ICIs in the context of a complex TME is strongly suggested to improve current effectiveness and design more efficient therapies.

Immune-infiltrating cells are essential among the multiple components of the TME and they play dual roles of tumour promotion and inhibition. Here, we calculated fractions of 22 types of immune cells and found that patients with BCa could be classified into subtypes C1, C2, and C3. The C1 subtype was enriched in CD8^+^ and activated CD4^+^ memory T cells, activated NK cells and M1 macrophages. The C2 subtype was enriched with activated mast cells and M0 macrophages, whereas the C3 subtype was enriched with monocytes, B cells, and resting CD4^+^ memory T cells, and resting dendritic cells. Survival was better for the C1, than the other two subtypes. In the TME CD8^+^ T cells play an anti-tumor role by recognising MHC I molecules, and patients with BCa and more abundant CD8^+^ T cells have better survival^18^. The Th1, Th2, Th9, Th17, and Treg subsets of CD4^+^ T cells are associated with a better prognosis of BCa^19,20^. The mechanisms through which T cells and their subtypes orchestrate cancer immunity are not clear, but T-cell antigen receptor (TCR) signalling does play an important role in T-cell activation and trafficking to the TME. For example, CSK negatively regulates T cell activation by repressing the initiation of TCR signalling, which correlates with ICI resistance^21^. Therefore, ICI immunotherapy might be improved by targeting TCR signalling. M1 and M2 macrophages with extreme phenotypes are essential components of the TME. M1 macrophages inhibit tumour development, progression, and angiogenesis, whereas M2 macrophages promote the initiation, growth, and progression of tumors^22^. The prognosis of patients with enhanced numbers of antitumor immune cells was improved in the present study; this further emphasised the importance of infiltrative immune-cell profiles in the progression and survival of patients with BCa.

Immune checkpoint inhibitors comprise novel therapeutic approaches for patients with BCa, but only a few respond to them^23^. The TME is closely associated with response rates to ICIs. For example, extant T-cell immunity correlates with responses to anti-PD-L1 therapy^24^. High antitumor T cell infiltration and macrophage polarisation are closely correlated with higher response rates^24,25^. M1 infiltration can predict immunotherapeutic responses among patients with BCa ^26^. Our results are consistent with these. Moreover, the C1 subtype with the greater TMB had the highest response rate, which confirmed that responders have higher TMBs^27^. Notably, PD-L1 expression on ICs but not on TCs, is associated with a response^11^. Here, we found that that the response rate was lower in the C3 subtype, which expressed the least amount of PD-L1 in tumour cells. This suggested that the role of PD-L1 expressed in tumour cells requires further exploration. Our findings showed that abnormal metabolism such as fatty acid degradation, glycolysis, and the PI3K-Akt pathways might reduce response rates. Targeting glycolysis is the most frequently explored strategy to improve immunotherapy., Abundant lactate dehydrogenase (LDH) is associated with a poor response of melanoma to anti-PD-1 therapy^28^. Increased glycolytic activity can impair the response to anti-PD-1 immunotherapy by inhibiting T cell killing^29^. Diclofenac, which inhibits glycolysis, also enhances anti-PD-1 immunotherapy^30^. Based on current findings, targeting glycolysis is a promising strategy for increasing ICI effectiveness. In addition, ECM-related pathways might also hijack tumoricidal immunity, and the ECM could modulate the activity of T cells and macrophages, thus influencing anti-tumor immune responses^31^. Elevated collagen can promote CD8^+^ T cell exhaustion through Leukocyte associated immunoglobulin like receptor 1 ***(***LAIR1) receptors and anti-PD-1 treatment, and a blockade of LAIR1 significantly reduces tumour growth and metastasis^32^. Therefore, a combination of ICIs and a blockade targeting these dysregulated pathways might change the TME landscape. This could enhance antitumor potential, increase sensitivity to immunotherapy, and thus might be a promising treatment for non-responders.

The differentiation and function of immune cells in the TME are intrinsically linked to metabolic alterations, and metabolic reprogramming has profound effects on tumourigenesis, development, and progression^33^. The competition for substrates between tumour and immune cells remodels the TME to become immunosuppressive or immune activated^34^. Cells need to produce energy to maintain homeostatic processes, while also meeting the demands of synthesising necessary macromolecules. We examined the metabolic profiles of the C1, C2, and C3 subtypes. We found that the metabolic profiles of C1 and C2 were similar and distinctly differed from that of the C3 subtype. Most lipid metabolism-related pathways and some important carbohydrate metabolism-related pathways, such as glycolysis/ gluconeogenesis, were enriched in the C3 subtype. Nucleotide metabolism was enriched in the C1 and C2 subtypes. Lipid metabolic remodelling, characterised by increased lipid uptake, storage, and lipogenesis, is a hallmark of cancer^35^. Tumours with lymph node metastasis accumulate more FAs as fuel and undergo a metabolic shift toward fatty acid oxidation^36^. Moreover, tumour cells can reprogram the physiology of adipocytes, stimulating them to reach distant organs^37^. These altered lipid metabolic pathways promote tumour cell migration and progression. Among altered metabolic programs, aerobic glycolysis (Warburg effect), has received the most attention^38^. The upregulation of glycolytic genes is directly related to BCa initiation and progression^39^. For example, the primary glucose uptake transporter-1 (GLUT1) is upregulated in tumours compared with normal tissues, and this correlates with poor cause-specific and overall survival^40^. The present findings showed that activated cells were enriched in the C1 subtype, whereas resting cells were mainly enriched in C3 subtype. The Warburg effect in tumor cells reduces lactate accumulation, which suppresses the activity of antitumor cells, including CD8^+^ T and NK cells^41,42^. The competitive TME impairs the activation of immune cells by limiting their glycolytic capacity. Taken together, these altered metabolic pathways in the TME of BCa might result in distinct levels and types of immune cells in different subtypes, and directly or indirectly result in the variable outcomes associated with BCa.

We developed a TME gene-related signature to predict the survival of patients with BCa and anti-PD-L1 response rates. Although PD-L1 expression is associated with response rates, its potential as a therapeutic response predictor has not been established^43^. The predictive ability of our signature exceeded that of PD-L1 mRNA expression and immunohistochemistry findings in ICs. We found higher response rates among high-risk patients with BCa. Therefore, anti-PD-L1 therapy should be recommended for this type of patient. This would help to develop personalised approaches for patients with BCa. However, this study has several limitations. For example, we applied only bioinformatic methods to reveal the heterogeneity of BCa. Therefore, more large-scale investigations in vivo and in vitro are needed to confirm our findings.

## Conclusions

We analysed infiltration profiles of immune cells and revealed molecular features associated with response rates of three subtypes of BCa. We also developed a model that can predict OS and anti-PD-L1 responses in patients with BCa.

### Supplementary Information

Supplementary Figures.

## Data Availability

All data in present study originated from the public database, the TCGA database (https://portal.gdc.cancer.gov/) and GEO database GSE13507, https://www.ncbi.nlm.nih.gov/geo/). The expression profile of IMvigor210 cohort was extracted from the IMvigor210CoreBiologies packages.

## References

[1] Bray F (2018). Global cancer statistics 2018: GLOBOCAN estimates of incidence and mortality worldwide for 36 cancers in 185 countries. CA Cancer J. Clin..

[2] Berdik C (2017). Unlocking bladder cancer. Nature.

[3] Chavan S, Bray F, Lortet-Tieulent J, Goodman M, Jemal A (2014). International variations in bladder cancer incidence and mortality. Eur. Urol..

[4] Cao R, Yuan L, Ma B, Wang G, Tian Y (2021). Tumour microenvironment (TME) characterization identified prognosis and immunotherapy response in muscle-invasive bladder cancer (MIBC). Cancer Immunol. Immunother..

[5] Annels NE, Simpson GR, Pandha H (2020). Modifying the non-muscle invasive bladder cancer immune microenvironment for optimal therapeutic response. Front. Oncol..

[6] Francisco LM (2009). PD-L1 regulates the development, maintenance, and function of induced regulatory T cells. J. Exp. Med..

[7] Okazaki T, Honjo T (2006). The PD-1-PD-L pathway in immunological tolerance. Trends Immunol..

[8] Li X, Shao C, Shi Y, Han W (2018). Lessons learned from the blockade of immune checkpoints in cancer immunotherapy. J. Hematol. Oncol..

[9] Rotte A, Jin JY, Lemaire V (2018). Mechanistic overview of immune checkpoints to support the rational design of their combinations in cancer immunotherapy. Ann. Oncol..

[10] Inman BA, Longo TA, Ramalingam S, Harrison MR (2017). Atezolizumab: A PD-L1-blocking antibody for bladder cancer. Clin. Cancer Res..

[11] Mariathasan S (2018). TGFbeta attenuates tumour response to PD-L1 blockade by contributing to exclusion of T cells. Nature.

[12] Zeng D (2021). IOBR: Multi-omics immuno-oncology biological research to decode tumor microenvironment and signatures. Front. Immunol..

[13] Mayakonda A, Lin DC, Assenov Y, Plass C, Koeffler HP (2018). Maftools: Efficient and comprehensive analysis of somatic variants in cancer. Genome Res..

[14] Newman AM (2019). Determining cell type abundance and expression from bulk tissues with digital cytometry. Nat. Biotechnol..

[15] Wilkerson MD, Hayes DN (2010). ConsensusClusterPlus: A class discovery tool with confidence assessments and item tracking. Bioinformatics.

[16] Wu T (2021). clusterProfiler 4.0: A universal enrichment tool for interpreting omics data. Innovation (N Y).

[17] Benitez JC, Remon J, Besse B (2020). Current panorama and challenges for neoadjuvant cancer immunotherapy. Clin. Cancer Res..

[18] Sharma P (2007). CD8 tumor-infiltrating lymphocytes are predictive of survival in muscle-invasive urothelial carcinoma. Proc. Natl. Acad. Sci. USA.

[19] Ben Khelil M (2022). Harnessing antitumor CD4 T cells for cancer immunotherapy. Cancers.

[20] Jiang A (2020). The construction and analysis of tumor-infiltrating immune cells and ceRNA networks in bladder cancer. Front. Genet..

[21] Sridaran D (2022). Inhibiting ACK1-mediated phosphorylation of C-terminal Src kinase counteracts prostate cancer immune checkpoint blockade resistance. Nat. Commun..

[22] Leblond MM, Zdimerova H, Desponds E, Verdeil G (2021). Tumor-associated macrophages in bladder cancer: Biological role, impact on therapeutic response and perspectives for immunotherapy. Cancers (Basel).

[23] Mancini M, Righetto M, Noessner E (2021). Checkpoint inhibition in bladder cancer: Clinical expectations, current evidence, and proposal of future strategies based on a tumor-specific immunobiological approach. Cancers (Basel).

[24] Chen X (2021). Analysis of tumor microenvironment characteristics in bladder cancer: Implications for immune checkpoint inhibitor therapy. Front. Immunol..

[25] Sun M (2021). Infiltration and polarization of tumor-associated macrophages predict prognosis and therapeutic benefit in muscle-invasive bladder cancer. Cancer Immunol. Immunother..

[26] Zeng D (2020). Macrophage correlates with immunophenotype and predicts anti-PD-L1 response of urothelial cancer. Theranostics.

[27] Rosenberg JE (2016). Atezolizumab in patients with locally advanced and metastatic urothelial carcinoma who have progressed following treatment with platinum-based chemotherapy: A single-arm, multicentre, phase 2 trial. Lancet.

[28] Hodi FS (2018). Nivolumab plus ipilimumab or nivolumab alone versus ipilimumab alone in advanced melanoma (CheckMate 067): 4-year outcomes of a multicentre, randomised, phase 3 trial. The Lancet. Oncology.

[29] Renner K (2019). Restricting glycolysis preserves T cell effector functions and augments checkpoint therapy. Cell Rep..

[30] Gottfried E (2013). New aspects of an old drug–diclofenac targets MYC and glucose metabolism in tumor cells. PloS One.

[31] Rømer AMA, Thorseth M-L, Madsen DH (2021). Immune modulatory properties of collagen in cancer. Front. Immunol..

[32] Peng DH (2020). Collagen promotes anti-PD-1/PD-L1 resistance in cancer through LAIR1-dependent CD8 T cell exhaustion. Nat. Commun..

[33] Pavlova NN, Thompson CB (2016). The emerging hallmarks of cancer metabolism. Cell Metab..

[34] Renner K (2017). Metabolic hallmarks of tumor and immune cells in the tumor microenvironment. Front. Immunol..

[35] Cheng C, Geng F, Cheng X, Guo D (2018). Lipid metabolism reprogramming and its potential targets in cancer. Cancer Commun. (Lond).

[36] Lee CK (2019). Tumor metastasis to lymph nodes requires YAP-dependent metabolic adaptation. Science.

[37] Dirat B (2011). Cancer-associated adipocytes exhibit an activated phenotype and contribute to breast cancer invasion. Cancer Res..

[38] Vander HMG, Cantley LC, Thompson CB (2009). Understanding the Warburg effect: The metabolic requirements of cell proliferation. Science New York (N.Y.).

[39] Woolbright BL, Ayres M, Taylor JA (2018). Metabolic changes in bladder cancer. Urol. Oncol..

[40] Hoskin PJ, Sibtain A, Daley FM, Wilson GD (2003). GLUT1 and CAIX as intrinsic markers of hypoxia in bladder cancer: Relationship with vascularity and proliferation as predictors of outcome of ARCON. Br. J. Cancer.

[41] Fischer K (2007). Inhibitory effect of tumor cell-derived lactic acid on human T cells. Blood.

[42] Husain Z, Huang Y, Seth P, Sukhatme VP (2013). Tumor-derived lactate modifies antitumor immune response: Effect on myeloid-derived suppressor cells and NK cells. J. Immunol..

[43] Song D (2019). Bladder cancer, a unique model to understand cancer immunity and develop immunotherapy approaches. J. Pathol..

