# Supplementary material for: Tumor microenvironment heterogeneity in bladder cancer identifies biologically distinct subtypes predicting prognosis and anti-PD-L1 responses
Source: Sci Rep. 2023 Nov 10;13:19563. doi: 10.1038/s41598-023-44028-3 (PMC10638294; doi:10.1038/s41598-023-44028-3)
Supplement: Supplementary file 1 — Supplementary Figures. [file 41598_2023_44028_MOESM1_ESM.pdf]

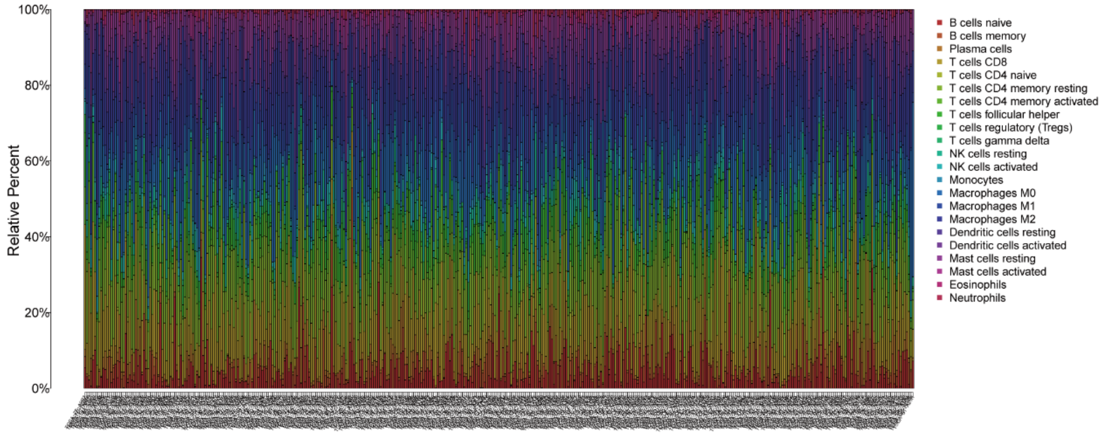

Supplementary Figure 1: The barplot of 22 kinds of immune cells in the TCGA-BLCA cohort.

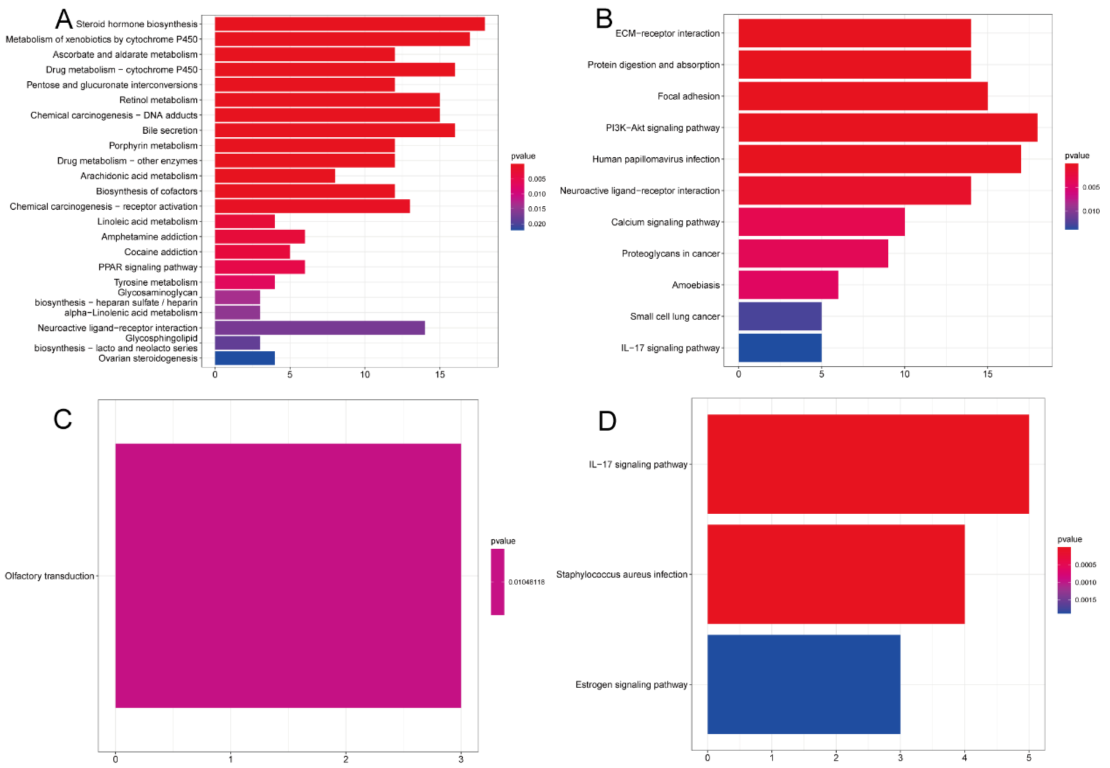

Supplementary Figure 2: Function enrichment analysis. KEGG enrichment analysis of genes in the blue (A), red (B), green (C), and brown modulate (D).

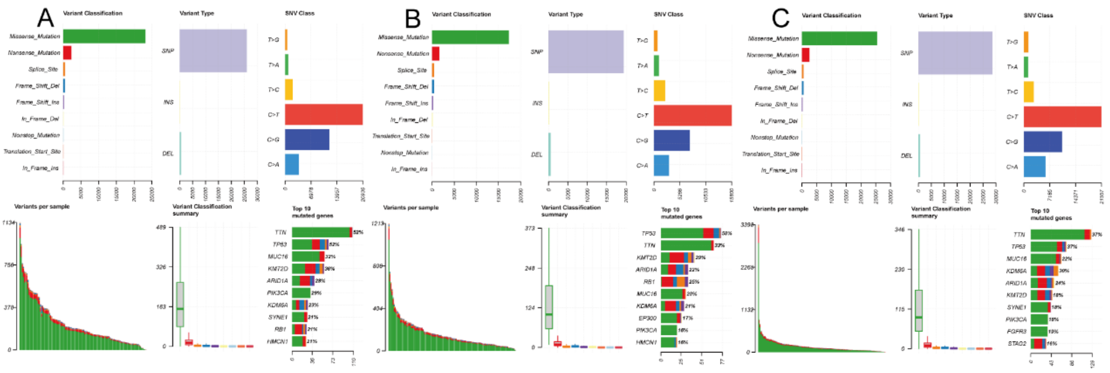

Supplementary Figure 3: Landscape of mutation profiles in the three subtypes of BCa. Summary of the mutation information in C1 subtype (A), C2 subtype (B), and C3 subtype (C).
